# Compound heterozygous variants of the *NARS2* gene in siblings with refractory seizures: two case report and literature review

**DOI:** 10.3389/fped.2025.1571426

**Published:** 2025-04-08

**Authors:** Heyan Wu, Min Zhou, Xiaoting Ye, Huabao Chen, Hongxin Lin, Li Wang, Xing Nie, Lidan Zhang

**Affiliations:** ^1^ Pediatric Intensive Care Unit, The Seventh Affiliated Hospital of Sun Yat-sen University, Shenzhen, China; ^2^ Department of Pediatrics, The Seventh Affiliated Hospital of Sun Yat-sen University, Shenzhen, China

**Keywords:** combined oxidative phosphorylation deficiency 24 (COXPD24), NARS2, refractory epilepsy, biallelic variants, pediatric, mitochondrial drug cocktail therapy

## Abstract

**Background:**

Biallelic variants in *NARS2* that encodes the mitochondrial asparaginyl-tRNA synthetase are associated with a wide spectrum of clinical phenotypes. Herein, we report on two siblings carrying the same compound heterozygous missense variants in *NARS2*, to improve the understanding of the phenotypic heterogeneity of *NARS2* variants.

**Case presentation:**

The two probands, a 3-year-old female (Patient 1) and a 16-month-old male (Patient 2), were clinically suspected of Combined oxidative phosphorylation deficiency 24 (COXPD24). Both presented with neurological manifestations, including refractory epilepsy, developmental delay and motor developmental regression, within the first year of life, accompanied by symmetrical brain lesions identified on magnetic resonance imaging (MRI). To elucidate the underlying genetic etiology, whole-exome sequencing (WES) was performed, followed by Sanger sequencing validation in the patients and their non-consanguineous parents. Genetic analysis revealed that both probands harbored identical compound heterozygous variants in the *NARS2* gene: c.1253G>A (p.Arg418His) and c.1163C>T (p.Thr388Met). Notably, the c.1163C>T (p.Thr388Met) variant in *NARS2* represents a novel finding, further expanding the genetic spectrum associated with this disorder.

**Conclusions:**

Our findings expand the mutational spectrum of *NARS2* and highlight the associated phenotypic heterogeneity, underscoring the critical role of *NARS2* in epilepsy and neurodevelopmental processes. For pediatric patients with refractory epilepsy, early genetic testing is essential to improve diagnostic accuracy, refine prognostic stratification, and guide personalized treatment strategies. Additionally, mitochondrial drug cocktail therapy may be beneficial for epilepsy caused by NARS2 mutations.

## Introduction

Mitochondrial diseases encompass a broad spectrum of disorders, arising from genetic defects that impair mitochondrial function, leading to ATP synthesis impairment and subsequent energy depletion (1). Although various possible clinical phenotypes can result, neurological and neuromuscular affection is most frequently encountered. Among these complex conditions, refractory epilepsy stands as a prominent and challenging aspect, intricately intertwined with mitochondrial dysfunction (2). On 11q14.1, *NARS2* encodes mitochondrial aminoacyl-tRNA synthetases (mt-ARSs), which catalyzes the ligation of asparagine to tRNA molecules and is essential for protein synthesis (3). While *NARS2* is widely expressed in human tissues, variants in the gene seem to preferentially affect tissues with high energy demand, such as the brain, cochlea and muscle, similar to other mitochondrial disorders (4). Biallelic variants in *NARS2* have been linked to a spectrum of clinical phenotypes, ranging from isolated hearing loss to severe neurodevelopmental disorders, with well-known examples being Combined oxidative phosphorylation deficiency 24 (COXPD24), Autosomal recessive deafness-94 (DFNB94), Alpers syndrome and Leigh syndrome. COXPD24 is an autosomal recessive disorder characterized by early in life with seizures, hypotonia, myopathy, hearing impairment, and overall delay and/or regression of cognitive and motor development (5–9). DFNB94 causes patients to exhibit bilateral nonsyndromic sensorineural hearing loss (10, 11). Despite its fundamental function, the full spectrum of NARS2-related disorders remains elusive due to the limited number of reported cases, the genotype-phenotype relationships remain unpredictable due to a limited number of known cases. Furthermore, the underlying mechanisms of NARS2-related disease have not been comprehensively studied. The purpose of this report is to provide detailed clinical, laboratory, and imaging data for two siblings carrying the same heterozygous missense mutations in the NARS2 gene, so as to enhance the understanding of the phenotypes of NARS2 gene variants.

## Case presentation

**Figure 1:**
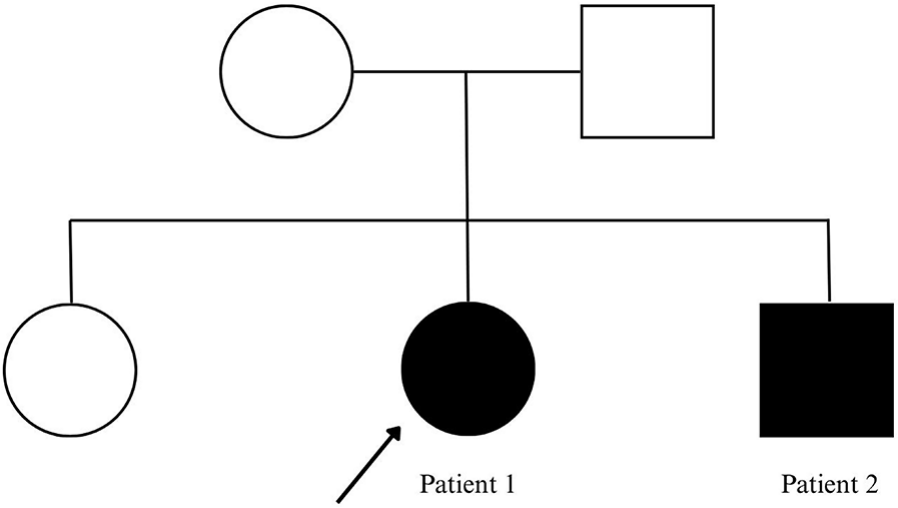
Family pedigree. Probands with status epilepticus are marked by filled symbols and arrows.

The two probands were born to non-consanguineous parents, as shown in Figure 1. Notably, their eldest sister showed no clinical manifestations of the condition.

**Figure 2:**
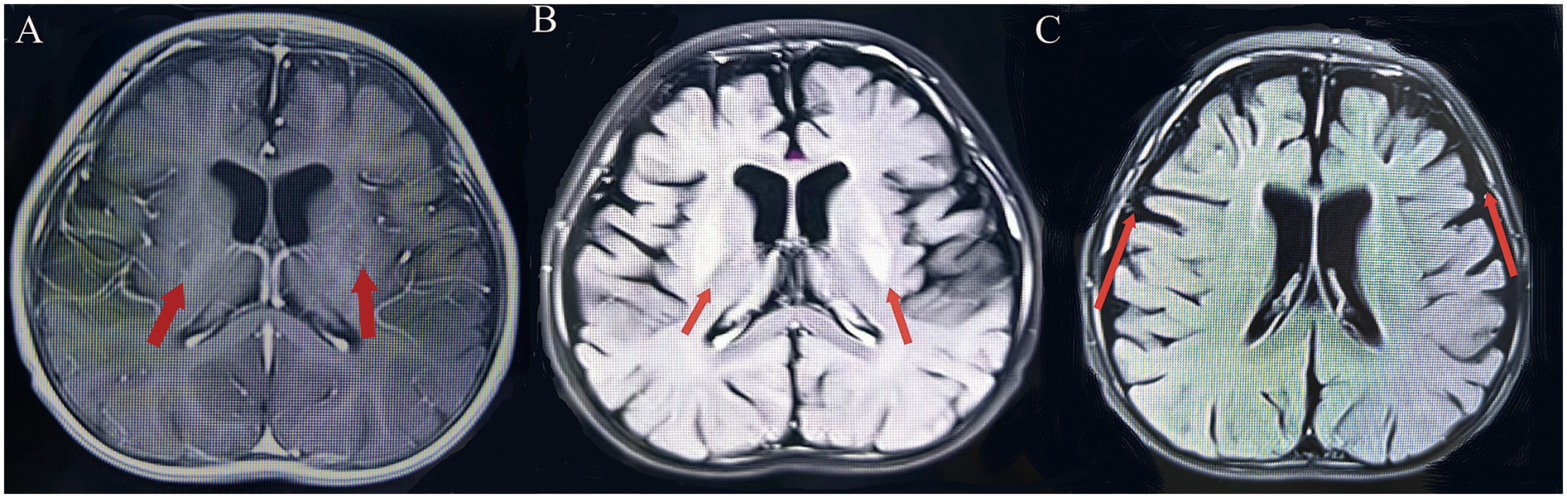
(Patient 1) Brain MRI. **(A)** T1-weighted image: symmetrical lenticular nucleus hyperintensity. **(B)** T2-weighted image: symmetrical lenticular nucleus hyperintensity. **(C)** Diffuse cerebral atrophy.

Patient 1: a 3-year-old girl is the second child of healthy nonconsanguineous Chinese parents. She was delivered at term without asphyxia. The infant exhibited typical developmental progress until reaching 6 months of age, at which point generalized epilepsy with myoclonic seizures activity manifested. A subsequent episode of convulsions occurred 1 month later. Following a third instance of seizures at 8 months, the child experienced a decline in developmental milestones, accompanied by diminished muscular strength and reduced muscle tone. The MRI performed at 8 months of age indicated delayed myelination, symmetrical abnormal signals in the lentiform nucleus, and diffuse cerebral atrophy (Figure 2). The electroencephalogram (EEG) examination revealed an abnormal pattern, characterized by the presence of a significant number of spike-and-slow-wave complexes in the bilateral occipital regions. Unfortunately, as the test was conducted at an external hospital, we were unable to obtain the EEG images. Topiramate tablets were administered as a means to curb the progression of epilepsy in this child. Despite titrating topiramate tablets to the maximum tolerated dose, the child continued to exhibit progression of seizures and developed abnormal liver function, leading to the decision to discontinue the antiepileptic medication. To minimize the risk of withdrawal symptoms, a structured tapering regimen was implemented, reducing the dosage by 25% every two weeks, with close monitoring for seizure recurrence and potential adverse effects. This approach was designed to ensure a safe and controlled discontinuation process while prioritizing the patient's clinical stability. Subsequently, there was a gradual decrease in both the frequency and intensity of epileptic seizures, though partial onset seizures persisted. As the condition advanced, the child eventually progressed into flaccid quadriplegia by the age of two. Despite the recommendation for a muscle biopsy as a diagnostic measure, the parents opted against the invasive procedure. In an effort to unravel the underlying pathology, Whole Exome Sequencing (WES) was undertaken for both the child and her family members. The whole exon detected two novel compound heterozygotes variants: NM_024678.6:c.1253G>A(p.Arg418His) mutation (from father) and NM_024678.6:c.1163C>T(p.Thr388Met) mutation (from mother) in *NARS2* gene, diagnosed with COXPD24.

**Figure 3:**
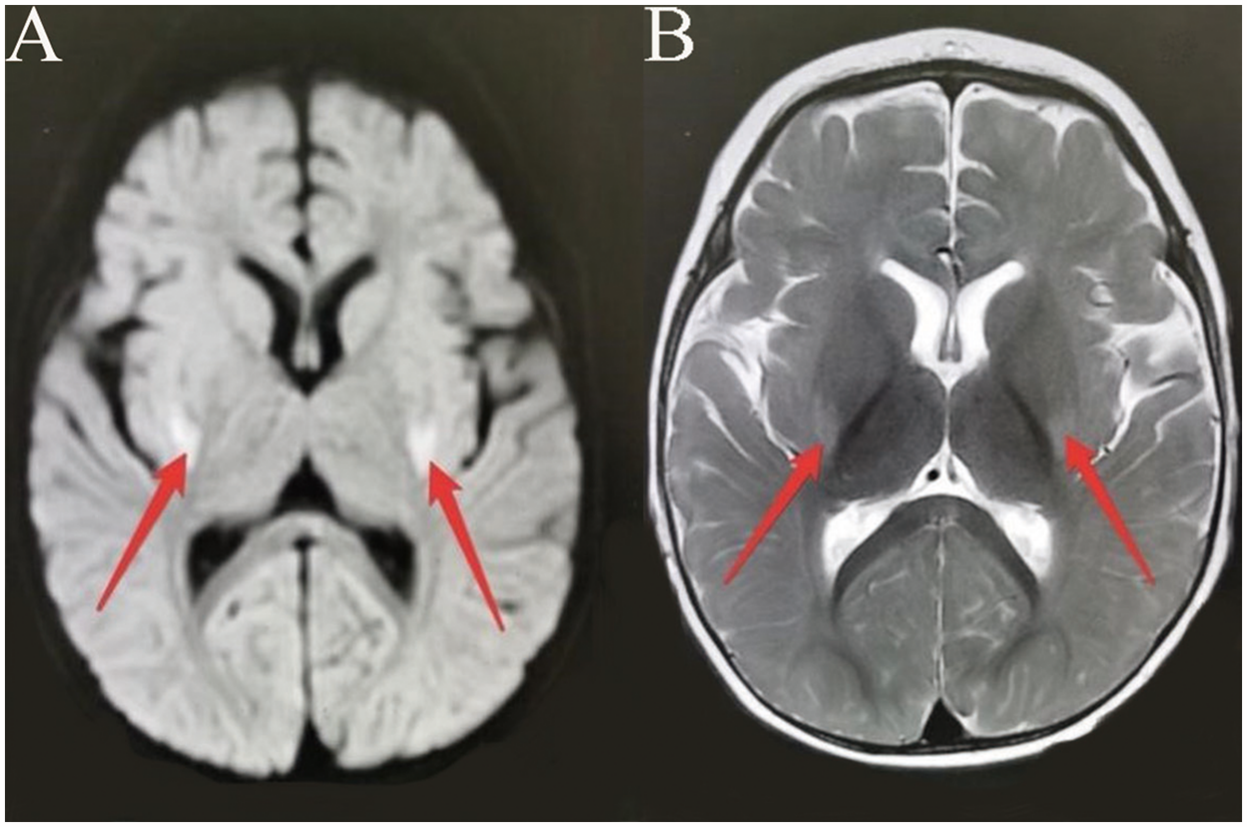
(Patient 2) Brain MRI. **(A)** T1-weighted image: bilateral posterior putamen hyperintensity. **(B)** T2-weighted image: bilateral posterior putamen hyperintensity.

**Figure 4:**
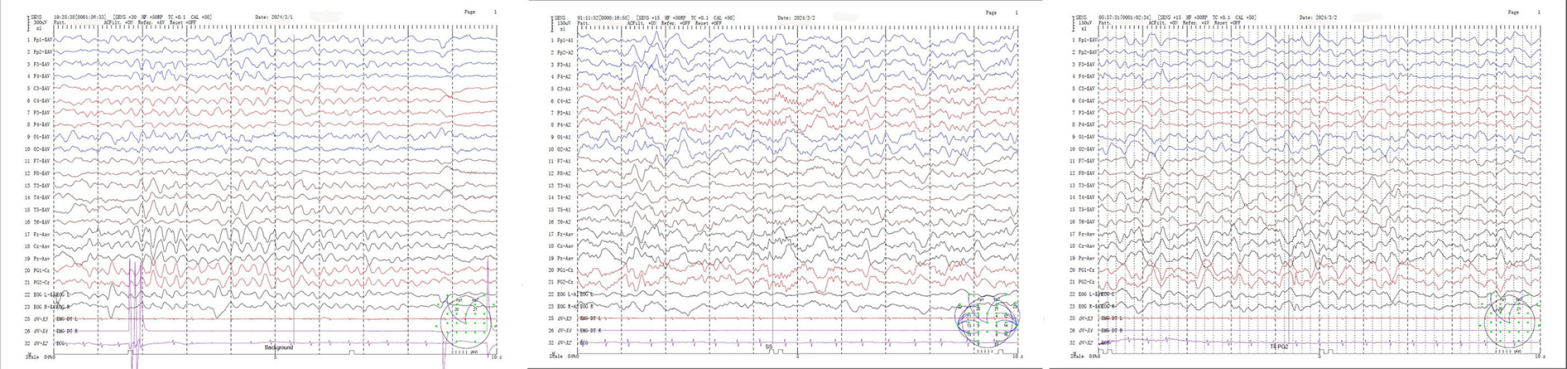
(Patient 2) EEG: slow background activity.

Patient 2, a 14-month-old boy and the younger brother of Patient 1, had an uneventful birth history and showed normal growth and development until the onset of his condition. At 6 months old, he achieved a developmental milestone by sitting unsupported. The patient was admitted to the hospital at 6 months of age due to seizures following a high fever. Upon physical examination upon admission, it was found that the patient was unable to concentrate or track objects, did not make eye contact, and was unresponsive to auditory stimuli. During the seizure episodes, the patient exhibited a tilt of the head and face to the right side and twitching of the right corner of the mouth, with the episodes lasting approximately 2 h before resolving spontaneously. During the hospital stay, the patient experienced 2 seizure episodes, characterized by a tilt of the head and face to the right side and twitching of the right corner of the mouth. Despite initial efforts to manage the seizures with oral chloral hydrate, the treatment proved ineffective, prompting the initiation of Levetiracetam therapy for seizure control. However, even with this intervention, occasional seizures persisted. Brain MRI imaging revealed a crucial clue: symmetrical abnormal signals in the bilateral posterior putamen (Figure 3). Additionally, EEG demonstrated a marked abnormality, featuring a slowed background rhythm and a proliferation of diffuse slow waves throughout the brain's conduction pathways (Figure 4). During his hospital stay, it became evident that Patient 2's motor development had regressed, making it difficult for him to maintain steady head control. Fortunately, other laboratory parameters, such as liver and cardiac enzyme levels, remained within normal limits. Genetic testing ultimately identified two mutations in the *NARS2* gene (c.1253G>A/p.Arg418His and c.1163C>T/p.Thr388Met), mirroring the findings in Patient 1, suggesting a familial link. Considering that this disease is a genetic disorder, we discontinued all antiepileptic drugs when the child was 8 months old and adopted a mitochondrial drug cocktail therapy (12), which included oral administration of coenzyme Q10 (5 mg/kg), L-carnitine(30 mg/kg), and vitamin B1 (10 mg/kg). Surprisingly, during the six—month follow—up, the child did not experience any further seizures.

**Figure 5:**
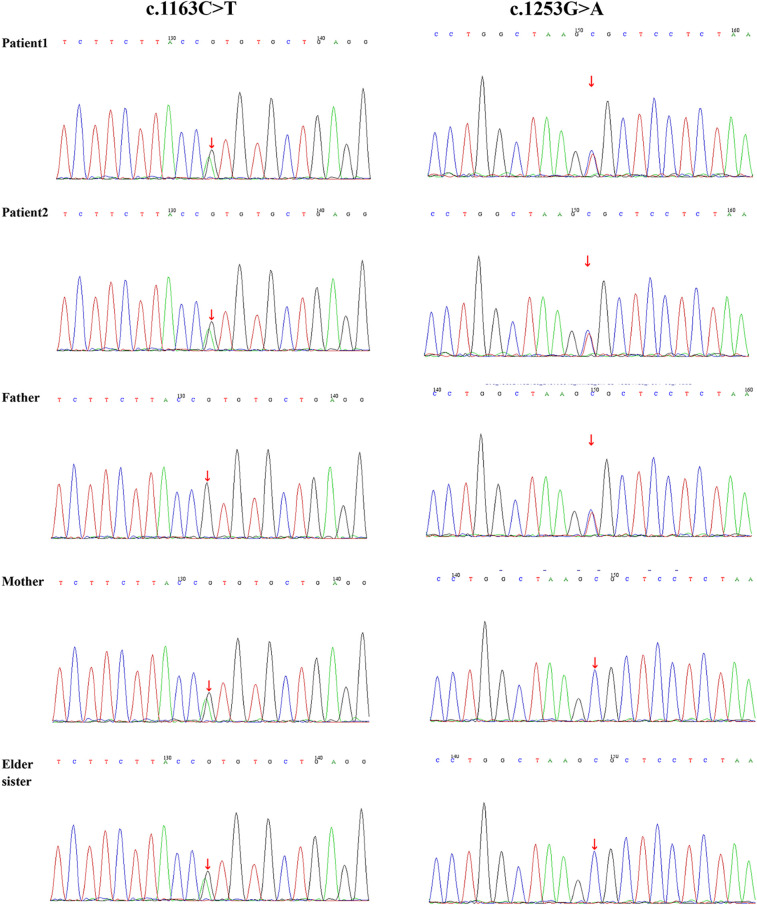
Sanger sequencing electropherograms validating the WES-identified variant in the proband.

To further explore the inheritance pattern and characteristics of these mutations, genetic verification was conducted on the healthy elder sister. The results revealed a unique genetic profile, as she carried only one of the two mutations identified in her siblings (c.1163C>T/p.Thr388Met), suggesting that a compound heterozygous mutation was necessary to cause the disease (Figure 5).

## Literature review

A comprehensive and thorough review of NARS2 mutations has been undertaken. As of the conclusion of December 2024, a total of 38 patients with a variant of NARS2 have been documented and reported. (Table 1) (5–11, 13–25), which included 32 missense variants, 3 truncation Missense, 2 splicing and 1 Synonymous. All the reported variants adhere to an autosomal recessive mode of inheritance, with their diagnosis, phenotypic manifestations, variant types, and survival outcomes comprehensively outlined in Table 1. The age of onset spans from the neonatal period to 50 years, with a notable trend towards early-onset disease. The phenotypic spectrum is notably heterogeneous, encompassing a range of conditions. Among the most salient phenotypic features previously reported in NARS2-related diseases are COXPD24, DFNB94, infantile-onset neurodegenerative disorder (NDD), and refractory seizures. Less frequently encountered manifestations include neonatal diabetes, microcephaly, and fatigability-associated ptosis. The disease outcomes vary greatly, with some patients passing away within a few weeks of birth, whereas others, particularly those with milder symptoms, reach adulthood. Nevertheless, based on the current genotype-phenotype correlation analysis, it remains challenging to accurately predict whether the phenotype will be mild or severe. Moreover, the research (13) has revealed that the phenotype does not appear to exhibit a substantial correlation with the location or type of mutation, underscoring the imperative need for the establishment of comprehensive genotype and phenotype databases. This underscores the importance of continued efforts to bridge the gap in our understanding of the intricate relationships between genetic variations and their phenotypic manifestations.

**Table 1:** Patients with mitochondrial disorders due to NARS2 variants reported as of the end of December 2024.

NARS2 mutation	Published time	Onset age	Survival outcome	Diagnosis	Variant type	References	Case no
c.822G>C; p.Q274H; Chr11(GRCh37):g.78204109C>G	2015	Not mention	Alive 34 years	COXPD24	Missense	Vanlander et al. (5)	1
c.822G>C; p.Q274H; Chr11(GRCh37):g.78204109C>G	2015	Childhood	Alive 26 years	COXPD24	Missense	Vanlander et al. (5)	2
c.969T>A;p.Tyr323* c.1142A>G;p.Asn381Ser	2015	Infantile	Deceased 15 months	Leigh Syndrome	Truncation Missense	Simon et al. (10)	3
c.969T>A;p.Tyr323* c.1142A>G;p.Asn381Ser	2015	Infantile	Deceased 6 months	Leigh Syndrome	Truncation Missense	Simon et al. (10)	4
c.637G>T; p.Val213Phe	2015	Not mention	Alive 45 years	DFNB94	Missense	Simon et al. (10)	5
c.637G>T; p.Val213Phe	2015	Not mention	Alive 40 years	DFNB94	Missense	Simon et al. (10)	6
c.637G>T; p.Val213Phe	2015	Not mention	Alive 26 years	DFNB94	Missense	Simon et al. (10)	7
c.637G>T; p.Val213Phe	2015	Not mention	Alive 30 years	DFNB94s	Missense	Simon et al. (10)	8
c.641C>T; p.Pro214Leu	2015	Perinatal	Deceased 16 years	Alpers syndrome	Missense	Sofou et al. (13)	9
c.707T>G;p.Phe236Cys c.594+1G>A;p.Asp172_Glu198del	2017	8 months	Alive 8 years	Infantile-onset neurodegenerative disorder	Missense	Mizuguchi et al. (6)	10
c.707T>G;p.Phe236Cys c.594+1G>A;p.Asp172_Glu198del	2017	10 months	Alive 1 years	Infantile-onset neurodegenerative disorder	Missense	Mizuguchi et al. (6)	11
c.151C>T; p.Arg51Cys c.1184T>G;p.Leu395Arg	2017	8 months	Alive 2 years	Infantile-onset neurodegenerative disorder	Missense	Mizuguchi et al. (6)	12
c.500A>G; p.His167Arg	2017	4 months	Alive 4 years	Infantile-onset neurodegenerative disorder	Missense	Mizuguchi et al. (6)	13
c.167A>G; p.Gln56Arg c.631T>A; p.Phe211Ile	2018	3 months	Deceased 6 months	COXPD24	Missense	Seaver et al. (7)	14
c.167A>G; p.Gln56Arg c.631T>A; p.Phe211Ile	2018	4 months	Deceased 6 months	COXPD24	Missense	Seaver et al. (7)	15
c.731C>G;p.Ala244Gly c.1351C>T;p.Arg451Cys	2020	Not mention	Not mention	Leigh syndrome	Missense	Lee et al. (14)	16
c.270C>T; p.Asn90Asn	2020	Perinatal	Alive 22 years	Reversible COX Deficiency	Synonymous	Palombo et al. (15)	17
c.641C>T; p.Pro214Leu	2021	Perinatal	Deceased 6 years	Alpers syndrome	Missense	Sofou et al. (16)	18
c.641C>T; p.Pro214Leu	2021	5 months	Alive 25 years	Alpers/Leigh syndrome	Missense	Sofou et al. (16)	19
c.545T>A; p.Ile182Lys	2021	12 months	Alive 17 years	COXPD24	Missense	Vafaee-Shahi et al. (21)	20
c.545T>A; p.Ile182Lys	2021	6 months	Alive 28 months	COXPD24	Missense	Vafaee-Shahi et al. (21)	21
c.83_84del;p.Leu28Glnfs*17 c.1339A>G;p.Met447Val	2021	3.5 months	Deceased 14 months	focal seizures; status epilepticus	Truncation missense	Sterbova et al. (17)	22
c.1141A>G;p.Asn381Asp c.1290G>C;p.Trp430Cys	2022	3 months	Deceased 6 months	COXPD24	Missense	Zhang et al. (8)	23
c.475C>T; p.Arg159Cys c.649T>G;p.Leu217Val	2022	3 months	Alive 3 years	Epilepsy; neonatal diabetes syndrome	Missense	Yagasaki et al. (22)	24
c.475C>T; p.Arg159Cys c.649T>G;p.Leu217Val	2022	Infantile	Alive 1 years	Epilepsy; neonatal diabetes syndrome	Missense	Yagasaki et al. (22)	25
c.1253G>A;p.Arg418His c.1300C>T;p.Leu434Phe	2022	Infantile	Alive 1 years	Leigh syndrome	Missense	Yang et al. (23)	26
c.556 A>G; p.Asn186Asp c.731C>G; p.Ala244Gly	2022	Infantile	Alive24 years	Leigh syndrome	Missense	Tanaka et al. (20)	27
c.506T>A; p.Phe169Tyr	2022	14 months	Alive 3 years	DFNB94	Missense	Al-Sharif et al. (11)	28
c.500 A>G; p.His167Arg	2022	Neonatal period	Alive 14 months	Type 1 diabetes;	Missense	Cokyaman et al. (19)	29
c.185T>C; p.Leu62Pro c.251+2T>G	2022	2 months	Deceased 11 months	Refractory epilepsy;epilepsia partialis developmental delay	Splicing	Hu et al. (9)	30
c.185T>C; p.Leu62Pro c.509T>G;p.Phe170Cys	2022	5 months	Alive 5 months	Refractory epilepsy; epilepsia partialis	Splicing	Hu et al. (9)	31
c.822G>C;p.Gln274His	2022	1 years	Alive 47 years	Hypoacusis	Missense	Ait-El-Mkadem et al. (18)	32
c.822G>C;p.Gln274His	2022	2 years	Alive 49 years	Hypoacusis; ataxia, tremor, spasticity	Missense	Ait-El-Mkadem et al. (18)	33
c.822G>C;p.Gln274His	2022	3 years	Alive 50 years	Hypoacusis; ataxia, tremor, spasticity	Missense	Ait-El-Mkadem et al. (18)	34
c.1352G>A; p.Arg451His c.707T>C; p.Phe236Ser	2023	3 months	Deceased 8 months	Epilepsy; developmental delay,	Missense	Yang et al. (25)	35
c.182C>T, c.446A>AG	2023	4.5 months	Alive 3.5 years	Developmental delay; intellectual disability; intellectual disability;	Missense	Finsterer and Mehri (24)	36
c.1253G>A/p.Arg418His c.1163C>T/p.Thr388Met		6 months	Alive 14 months	focal seizures regression of motor development	Missense	Our study	37
c.1253G>A/p.Arg418His c.1163C>T/p.Thr388Met		6 months	Alive 3 years	COXPD24	Missense	Our study	38

* Signifies a premature termination codon (PTC), resulting from a nonsense mutation.

## Discussion

We report a case of two siblings who carry identical compound heterozygous pathogenic mutations in the NARS2 gene, yet their prognoses are different because the treatment regimens applied to them vary. Both of them presented with neurological manifestations, such as refractory epilepsy, developmental delay, and motor developmental regression within the first year of life, while MRI showed symmetrical brain lesions and neither of them had impaired hearing. The slight difference was that the elder sister had generalized epilepsy with myoclonic seizures, while the younger brother had focal seizures. In our study, Patient 2 received prompt mitochondrial drug cocktail therapy shortly after the disease etiology was identified, which effectively controlled his epilepsy without the need for excessive antiepileptic drugs. Notably, compared to Patient 1, Patient 2 exhibited less pronounced neurodevelopmental regression. Mitochondrial cocktail therapy refers to a combination of supplements, such as coenzyme Q10, L-carnitine, and B vitamins, designed to enhance mitochondrial function, improve energy production, and reduce oxidative stress, primarily used in the treatment of mitochondrial and metabolic disorders (26–28). The positive response to mitochondrial drug cocktail therapy underscores the role of mitochondrial dysfunction in epileptogenesis, implicating NARS2 mutations as a key pathogenic mechanism. NARS2 encodes a mitochondrial aminoacyl-tRNA synthetase crucial for protein synthesis, and its mutations disrupt energy metabolism, leading to epilepsy. This therapeutic success further supports NARS2-related mitochondrial dysfunction as central to the disorder.

The c.1163C>T(p.Thr388Met) is a newly discovered variant in the NARS2 gene, which enrich our comprehension of the mutational spectrum of *NARS2*, enabling a more profound exploration into its intricacies. As invaluable additions to the gene mutation database, they lay a solid foundation for theoretical constructs that will underpin the development and implementation of precise gene therapeutic strategies in the future.

Previously, there had been report (23) of patient with the same mutation site, c.1253G>A (p.Arg418His), presenting with hyperlactatemia and myocardial dysfunction. However, our patient did not exhibit the same clinical phenotype. This striking demonstration of genetically identical variants resulting in different clinical manifestations underscores the intricate relationship between genotype and phenotype in NARS2-associated disorders, echoing previous observations in the literature (5). We posit that this phenotypic variability stems from a complex interplay of multiple factors, encompassing the specific nature and location of the mutations, as well as their differential effects on the functionality of tRNA synthetase. This finding is congruent with the intricate and multifaceted nature of other mitochondrial disorders, where genetic variations often lead to a diverse spectrum of clinical manifestations, underscoring the importance of nuanced and comprehensive genotype-phenotype analyses in these complex disease processes (29).

**Figure 6:**
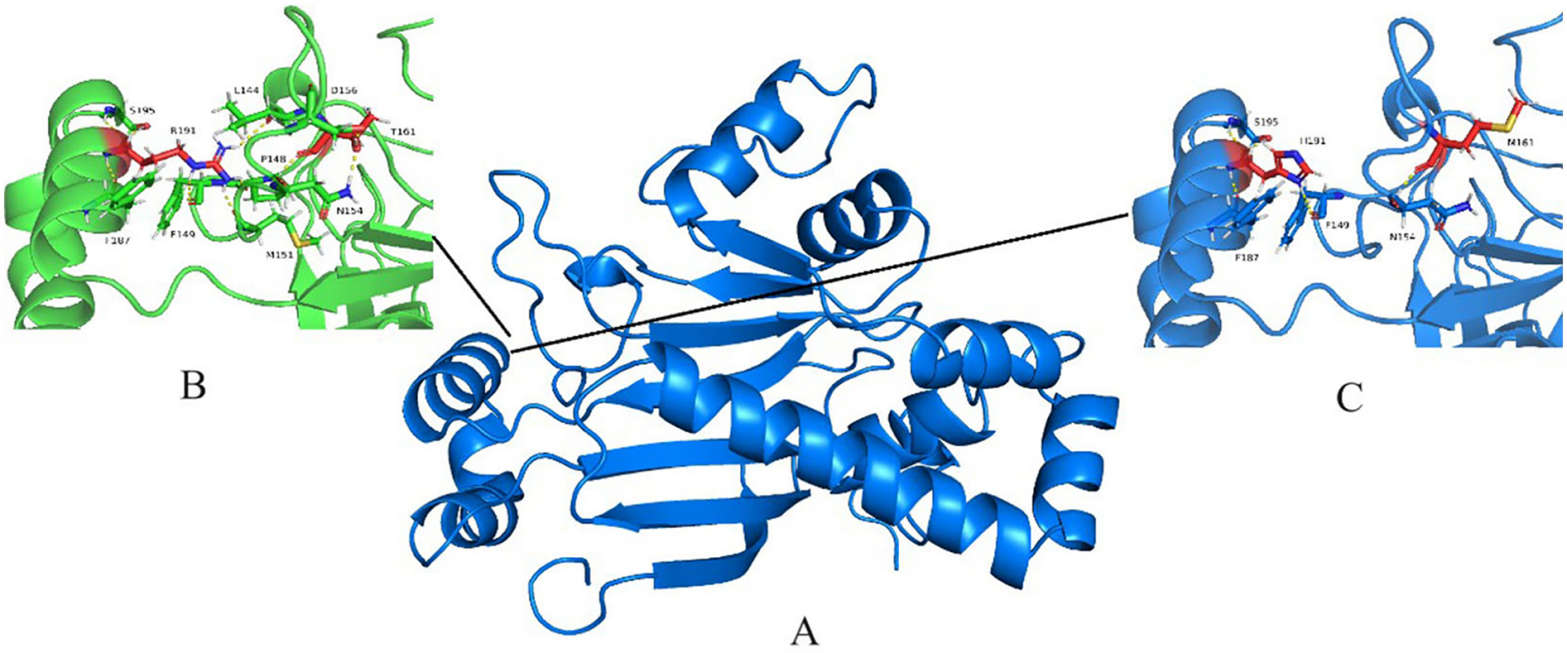
NARS2 protein structural modeling. **(A)** Alphafold2-predicted Asn-RS homodimer. **(B)** Close-up of wild-type region. **(C)** Mutated residues (yellow/green) in monomeric structure.

The *NARS2* encodes a protein of 477 amino acids that contains an anticodon binding domain (amino acids 44–118) and a catalytic domain (amino acids 135–472) of tRNA^Asn^ (http://www.uniprot.org/uniprot/). Our study identified two novel *NARS2* mutations, including the two missense mutations Arg418His and Thr388Met located in the catalytic domain (Figure 6). Although the comprehensive exploration of the pathophysiological implications of the detected compound heterozygous variants remains to be undertaken, parallels drawn from similar *NARS2* deficiencies hint at the likelihood that homozygous or compound heterozygous variants of *NARS2* lead to diminished enzyme synthesis, impaired translocation into mitochondria, compromised asparaginase ligation to tRNA molecules, and a cumulative oxidative phosphorylation insufficiency, collectively referred to as Combined Oxidative Phosphorylation Deficiency 24 (3). We posit that the mutation c.1253G>A, resulting in the amino acid substitution p.Arg418His within exon 12, involves a crucial shift from the phylogenetically conserved non-aromatic arginine (Arg) to the aromatic histidine (His). This transition is hypothesized to diminish the stability of the *NARS2* protein dimer through reduced binding free energy, with potential ramifications for the normal function of the *NARS2* enzyme in biological pathways. The variant c.1163C>T, leading to the amino acid substitution p.Thr388Met within exon 11, involves a transformation from the polar amino acid threonine to the non-polar methionine. This substitution may elicit a structural reconfiguration within the AsnRS homodimer, potentially altering its conformation and impacting the intricate arrangements necessary for optimal enzyme function. The PolyPhen program predicted the c.1253G>A and c.1163C>T mutation of *NARS2* to be “probably damaging” and the SIFT program anticipated these mutations to be “deleterious” (Table 2). Notably, these mutations were absent from any publicly accessible databases of single nucleotide polymorphisms (SNPs), further emphasizing their rarity and potential significance.

**Table 2:** NARS2 mutation information in this study.

Gene	NARS2	NARS2
Variant	c.1253G>A (p.Arg418His)	c.1163C>T (p.Thr388Met)
Chromosome	chr11:78154716	chr11:78176923
ExAC	0	0
GnomAD	0	0
SIFT	Deleterious	Deleterious
Mutation Taster	Damaging	Damaging
PolyPhen	Probably damaging	Probably damaging
ACMG rating	Uncertain significance	Uncertain significance
Rating evidence	PM2+PM3_Supporting	PM2+PM3

In this rigorous medical study, we have identified novel compound mutations in the *NARS2* gene that display a striking correlation with a diverse spectrum of clinical phenotypes. Although predictive computational tools, such as PolyPhen and SIFT, offer valuable preliminary assessments of the potential effects of these variants, definitive functional validation remains the gold standard for determining their mutational status. However, it is imperative to acknowledge certain limitations in our investigation. Due to the patient family's reluctance to undergo invasive diagnostic procedures, we were unable to directly assess mitochondrial respiratory chain function in patient-derived tissue specimens, thereby limiting the extent of our correlation analysis between the identified mutations and their underlying cellular functional consequences. Moreover, the lack of subcellular localization experiments and the inability to empirically verify the impact of these novel mutations on protein dimerization represent further constraints. Despite these limitations, the findings of this research represent a substantial contribution to our understanding of the mutational landscape of the *NARS2* gene and its intricate associations with a broad range of clinical presentations.

In conclusion, our study notably expands the phenotypic and genotypic spectrum of *NARS2* disorders through the identification of a novel disease-causing variant in two siblings. For pediatric refractory epilepsy, early genetic screening may expedite diagnosis and inform prognosis, facilitating personalized treatment. Additionally, to progress targeted therapies for NARS2-related diseases, intensified research is imperative to elucidate the intricate mechanisms underlying the complex effects of *NARS2* variants on mitochondrial protein synthesis.

## Data Availability

The datasets presented in this study can be found in online repositories. The names of the repository/repositories and accession number(s) can be found below: https://www.ncbi.nlm.nih.gov/, Bioproject accession number: PRJNA1170432.
